# Zika Virus Alters DNA Methylation of Neural Genes in an Organoid Model of the Developing Human Brain

**DOI:** 10.1128/mSystems.00219-17

**Published:** 2018-02-06

**Authors:** Sylvie Janssens, Michael Schotsaert, Rahul Karnik, Vinod Balasubramaniam, Marion Dejosez, Alexander Meissner, Adolfo García-Sastre, Thomas P. Zwaka

**Affiliations:** a Huffington Center for Cell-Based Research in Parkinson's Disease, Black Family Stem Cell Institute, Department of Cell, Developmental and Regenerative Biology, Icahn School of Medicine at Mount Sinai, New York, New York, USA; b Department of Microbiology, Global Health and Emerging Pathogens Institute, and Department of Medicine, Division of Infectious Diseases, Icahn School of Medicine at Mount Sinai, New York, New York, USA; c Harvard Stem Cell Institute, Department of Stem Cell and Regenerative Biology, Harvard University, and Broad Institute of MIT and Harvard, Cambridge, Massachusetts, USA; University of Chicago

**Keywords:** DNA methylation, Zika virus, astrocytes, brain disorders, microcephaly, neurons

## Abstract

Zika virus (ZIKV) infection during early pregnancy can cause microcephaly and associated defects at birth, but whether it can induce neurologic sequelae that appear later in life remains unclear. Using a model of the developing brain based on embryonic stem cell-derived brain organoids, we studied the impact of ZIKV infection on the DNA methylation pattern across the entire genome in selected neural cell types. The virus unexpectedly alters the DNA methylome of neural progenitors, astrocytes, and differentiated neurons at genes that have been implicated in the pathogenesis of a number of brain disorders, most prominently mental retardation and schizophrenia. Our results suggest that ZIKV infection during fetal development could lead to a spectrum of delayed-onset neuropsychiatric complications.

**IMPORTANCE** Scientific research on human neural stem cells and cerebral organoids has confirmed the congenital neurotropic and neurodestructive nature of the Zika virus. However, the extent to which prenatal ZIKV infection is associated with more subtle brain alterations, such as epigenetic changes, remains ill defined. Here, we address the question of whether ZIKV infection induces DNA methylation changes with the potential to cause brain disorders later in life.

## Abstract (precis)

Scientific research on human neural stem cells and cerebral organoids has confirmed the congenital neurotropic and neurodestructive nature of the Zika virus. However, the extent to which prenatal ZIKV infection is associated with more subtle brain alterations, such as epigenetic changes, remains ill defined. Here, we address the question of whether ZIKV infection induces DNA methylation changes with the potential to cause brain disorders later in life.

## INTRODUCTION

The Zika virus (ZIKV) appeared first in 1947 in Uganda and remained in tropical and subtropical ecologies in Africa and Asia, where it caused only mild symptoms in humans (1). In 2015, however, there was a large ZIKV outbreak in Brazil in which infection during pregnancy was clearly linked to severe anomalies, such as microcephaly in newborns (2), and thus galvanized the attention of the international biomedical community. The Brazilian epidemic also showed that adults who contracted the virus sometimes develop Guillain-Barré syndrome, while studies based on human *in vitro* models confirmed the neurotropic and neurodestructive potential of the Zika flavivirus (3–5). Still, ZIKV infection does not invariably produce disorders with overt manifestations (6), and the long-term pathological implications of such cases remain unclear.

Some toxins and pathogens alter fetal neurodevelopment, often by disrupting the epigenetic landscape of neuronal cells. For example, prenatal alcohol exposure and immune activation may induce epigenetic changes, which can manifest as neurologic diseases many years after the initial insult (7, 8). Similarly to toxins, viruses such as herpes simplex virus, cytomegalovirus, and Epstein-Barr virus can affect the epigenome as well (9, 10), but whether gestational ZIKV infection fits this category and could cause disorders of the brain later in life has yet to be elucidated.

## RESULTS

### DNA methylation changes after ZIKV infection.

We examined the effects of ZIKV infection on the epigenome using cerebral organoids as a model of the developing brain. This decision was based on several considerations. First, the generation of these models from human induced pluripotent stem cells (hiPSCs) leads to three-dimensional (3D) structures whose normal density is typical of the human brain and much higher than that found in other models (11). Second, the organoids, despite being an *in vitro* system, contain multiple organized brain tissues and possess a complexity that facilitates not only heterocellular interactions but also developmentally appropriate gene expression regulation and epigenetic patterning, thus closely mimicking the characteristic of human fetal neurodevelopment *in vivo* (12, 13). Third, this model allows us to simultaneously study cells at different phases of development, enabling us to test the crucial hypothesis that ZIKV induces methylation changes in neural progenitors, neurons, and glial cells.

**FIG 1:**
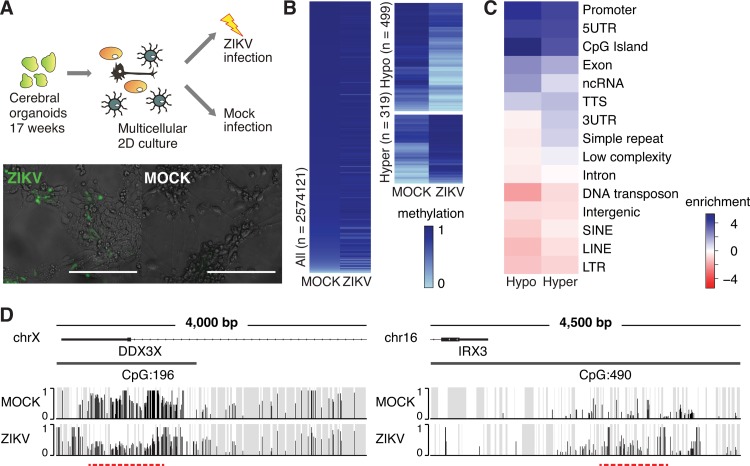
ZIKV infection induces global DNA methylation changes in multicellular human cerebral organoids. (A) (Top) Schematic of the samples used for whole-genome bisulfite sequencing (WGBS). (Bottom) ZIKV-positive cells (anti-flavivirus group E antigen, green) upon ZIKV infection (strain MR766) of cerebral organoid-derived 2D cultures. Bars, 100 µm. (B) Heat maps show methylation levels of all defined 1-kb tiles (left) and DMRs with an average methylation difference greater than 0.2 (right). (C) Enrichment [log_2_(hypergeometric *P* value)] of genomic features of differentially methylated 1-kb tiles (*q* value of <0.05 and methylation difference greater than 0.2). UTR, untranslated region; ncRNA, noncoding RNA; TTS, transcription termination site; SINE, short interspersed nuclear element; LINE, long interspersed nuclear element; LTR, long terminal repeat. (D) Examples of a hypomethylated (*DDX3X*) and hypermethylated (*IRX3*) gene locus. Gray, areas with nonsignificant sequencing signal; red dashed line, differentially methylated region.

To examine the effects of ZIKV on the epigenome, we cultured human embryonic stem cell (ESC)-derived organoids for as long as 16 weeks and confirmed their cortical nature by immunofluorescence staining with specific markers for neural progenitors, astrocytes, and neurons (see Fig. S1 in the supplemental material). We then disrupted the brain organoids—to enhance infection—and incubated them in 2D culture for 7 days before exposure to ZIKV (Uganda strain MR766; multiplicity of infection [MOI] of 1.0) or control medium for 72 h. Immunofluorescence staining to detect Zika viral proteins (E protein and NS5) corroborated infection of the brain organoid cultures and further showed in many instances nuclear localization of NS5 (Fig. 1A and S2A). The latter finding suggests that Zika virus components invade the nucleus of not only 293T cells (14) but also neural cells, providing a rationale for investigations into virus-associated epigenetic effects.

10.1128/mSystems.00219-17.1 FIG S1  Human ESC-derived cerebral organoid culture and detection of astrocytes, neurons, and neural progenitor cells. (A) Phase-contrast or bright-field (day 96) images of developing cerebral organoids. Different organoids are shown at different time points. Bar, 1,000 µm. (B) Phase-contrast microscopy of a 2D culture of 112-day-old organoids 5 days after dissociation shows different types of neuronal cells. Bar, 50 µm. (C) Immunofluorescence staining to detect astrocyte (GFAP, red), neuron (TUJ1, green), and neural progenitor (PAX6, red) markers in 16-week-old organoids. Bar, 100 µm. (D) Immunofluorescence staining to detect astrocyte (GFAP, red), neuron (DCX, red), and neural progenitor (PAX6, red) markers in organoid-derived dissociated multicellular cultures. Bar, 50 µm. Download FIG S1, DOCX file, 4.9 MB. Copyright © 2018 Janssens et al. 2018 Janssens et al. This content is distributed under the terms of the Creative Commons Attribution 4.0 International license.

10.1128/mSystems.00219-17.2 FIG S2  The 1947 Uganda and 2015 Puerto Rico ZIKV strains show comparable infection rates in human ESC-derived cerebral organoid cultures. (A) Immunofluorescence staining to detect the flaviviral E and NS5 proteins in multicellular 2D cultures derived from 84-day-old organoids infected with the Uganda (MR766) or Puerto Rico (PR) ZIKV strain. Bar, 10 µm. (B) Infection rates of 16-week-old cerebral organoid-derived astrocytes (astro), neural progenitor cells (NPC), and neurons (neuro) upon infection with the MR766 or PR ZIKV strain as determined by flow cytometry in two independent experiments. Download FIG S2, DOCX file, 0.8 MB. Copyright © 2018 Janssens et al. 2018 Janssens et al. This content is distributed under the terms of the Creative Commons Attribution 4.0 International license.

To test if the virus can indeed trigger methylation changes in our organoid model, we performed pilot experiments using whole-genome bisulfite sequencing (WGBS). By dividing the genome into 1-kb tiles and comparing the average methylation levels in each tile between ZIKV- and mock-infected samples, we found that many tiles showed differential methylation after exposure to ZIKV (Fig. 1B) and that those ZIKV-responsive genomic regions aligned well with epigenomic domains (13) found under physiological conditions in human fetal brain tissue (Fig. S3 and S4). Differentially methylated regions (DMRs) were defined as tiles that had an average difference of greater than 0.2 (weighted *t* test, false-discovery rate [*q* value] of <0.050). Among the 818 tiles in this analysis, 499 were hypomethylated and 319 were hypermethylated with strongholds in CpG islands, promoters, and exons but not enhancers (Fig. 1B to D). This methylation pattern suggests that ZIKV infection induces relatively specific effects on the epigenome, preferentially targeting regulatory regions around particular genes.

10.1128/mSystems.00219-17.3 FIG S3  ZIKV-sensitive DNA regions align well with regions of the fetal brain that are methylated. Visualization of methylation-sensitive restriction enzyme sequencing (MRE-seq) data obtained from fetal brain tissue at 17 weeks of gestation (NIH Epigenomics Roadmap Consortium, samples HuFNSC01 and HuFNSC02) aligned with ZIKV-induced differentially methylated regions (DMRs) identified in cerebral organoids by WGBS (1-kb tiles) in our study. Shown is the alignment of all human autosomes and the X chromosome (all analyzed samples are female). Download FIG S3, DOCX file, 2.7 MB. Copyright © 2018 Janssens et al. 2018 Janssens et al. This content is distributed under the terms of the Creative Commons Attribution 4.0 International license.

10.1128/mSystems.00219-17.4 FIG S4  Illustration of epigenetic marks in fetal brain gene regions that are ZIKV sensitive. Fetal brain epigenetic marks (at 17 weeks of gestation [NIH Epigenomics Roadmap Consortium]) are shown for the 200- to 250-kb region around the hypomethylated DDX3X locus (A) and the hypermethylated IRX3 locus (B) in ZIKV-infected cerebral organoids (also Fig. 1D). DMRs, differentially methylated regions; gray, areas with nonsignificant sequencing signal; red marks, differentially methylated regions. Download FIG S4, DOCX file, 1.2 MB. Copyright © 2018 Janssens et al. 2018 Janssens et al. This content is distributed under the terms of the Creative Commons Attribution 4.0 International license.

### ZIKV produces unique DNA methylation footprints in neural cell populations.

**FIG 2:**
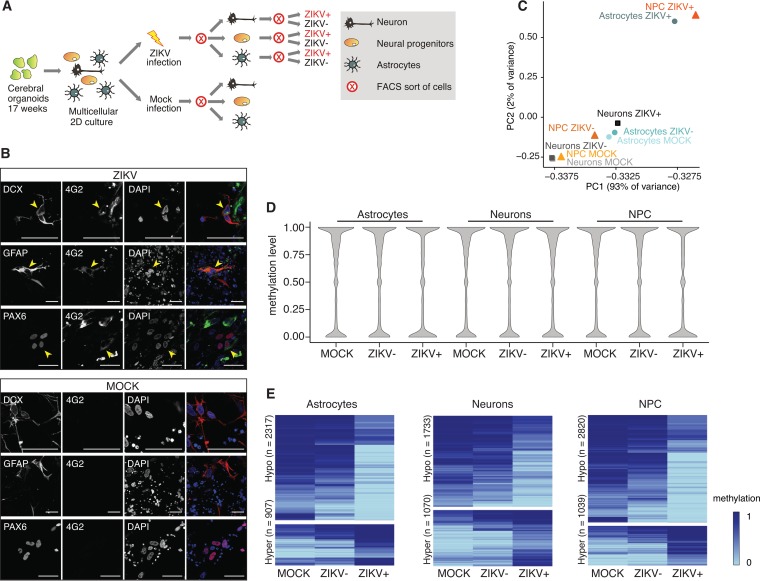
ZIKV infection induces DNA methylation changes in human cerebral organoid-derived astrocytes, neurons, and neural progenitor cells. (A) Schematic of the samples used for reduced representation bisulfite sequencing (RRBS). (B) Immunofluorescence staining of ZIKV- and mock-infected multicellular 2D cerebral organoid cultures to detect ZIKV (strain MR766) infection (4G2, anti-flavivirus group E antigen, green) in neurons (DCX, red), astrocytes (GFAP, red), and neural progenitor cells (PAX6, red). Bar, 100 µm. DAPI, 4′,6-diamidino-2-phenylindole. (C) Principal-component analysis based on the mean methylation levels of 100-bp tiles. (D) Distribution of methylation levels in samples with or without ZIKV (strain MR766) infection, as indicated. (E) Heat maps of differentially methylated 100-bp tiles in each cell type (*q* value of <0.05 and methylation difference greater than 0.2). NPC, neural progenitor cells.

Cerebral organoids harbor a number of promiscuous cell types (15), raising the possibility of conflated experimental results due to cellular diversity and the inability to exclude high noise levels of cell-type-specific DNA methylation signatures. We avoided this pitfall by isolating discrete cell populations from brain organoids by applying fluorescence-activated cell sorting (FACS) before DNA methylation analysis (Fig. 2A). Since ZIKV infects neural progenitor cells (NPCs), astrocytes, and neurons (4, 5, 16), as also seen in our organoids (Fig. 2B and S5B), we established FACS protocols to isolate each of these 3 cell types using their unique cell surface marker signatures (17) (Fig. S5A). To enhance the power of our analyses, we enriched for ZIKV-infected cells, by sorting against the flavivirus E antigen as an indicator of infection (Fig. S5B and C). To exclude the formal possibility that our results were virus strain specific, we performed additional inoculation studies using the Puerto Rico ZIKV strain and found infection rates similar to those seen with the Uganda strain (Fig. S2A and B).

10.1128/mSystems.00219-17.5 FIG S5  FACS strategy to segregate human ESC-derived cerebral organoids into astrocytes, neurons, and neural progenitor cells. (A) Examples of cell sorting plots and marker signatures (table) used to isolate astrocytes, neurons, and neural progenitor cells (NPC) from human ESC-derived cerebral organoid cultures. (B) Examples of FACS gating and percentages of ZIKV-infected cells among isolated astrocytes, neurons, and NPCs from infected (MR766) multicellular cerebral organoid cultures. (C) Scatter plot showing the distribution of astrocytes, neurons, and NPCs in ZIKV-infected (MR766) cerebral organoids in three independent experiments. The overall trend favors the presence of astrocytes (astro) and neural progenitors (NPC) over mature neurons (neuro) in the ZIKV^+^ cell fraction. Download FIG S5, DOCX file, 0.5 MB. Copyright © 2018 Janssens et al. 2018 Janssens et al. This content is distributed under the terms of the Creative Commons Attribution 4.0 International license.

**FIG 3:**
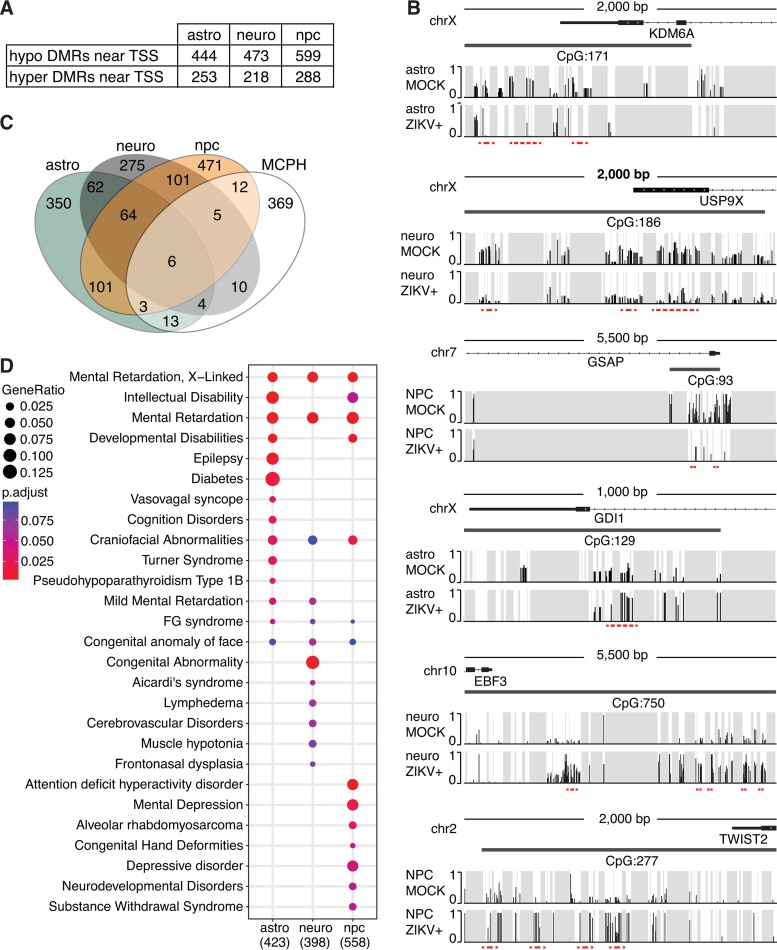
Disease ontology of genes associated with ZIKV-induced changes. (A) Numbers of hypomethylated and hypermethylated DMRs near gene loci (5,000 bp upstream to 500 bp downstream of transcription start sites [TSS]) per cell type. (B) Examples of hypomethylated (*KDM6A*, *USP9X*, and *GSAP*) and hypermethylated (*GDI1*, *EBF3*, and *TWIST2*) gene loci. Gray, areas with nonsignificant sequencing signal; red dashed line, differentially methylated region. (C) Venn diagram showing the number of affected gene loci in each cell type after ZIKV infection (strain MR766) and their overlap with genes defined by the human phenotype ontology for microcephaly (MCPH; total overlap of 53 out of 422 MCPH-related genes). (D) Dot plot of top DisGeNET disease categories that correlate with differentially methylated gene loci (5,000 bp upstream to 500 bp downstream of transcriptional start site). astro, astrocytes; neuro, neurons; npc, neural progenitor cells.

Given that, in our WGBS pilot studies, DMRs tended to be sequestered in or near promoters, we further assessed methylation of the sorted cell populations at CpG-rich regions by applying reduced representation bisulfite sequencing (RRBS) (18). The sequencing results were organized into 100-bp tiles, mapped, and subjected to principal-component analysis (Fig. 2C). The first two principal components (responsible for 95% of the variation among cell types) separated the ZIKV-positive from the mock-infected and ZIKV-negative cells, which clustered together closely according to cell type. We next compared methylation levels among the samples by density plotting (Fig. 2D). While there were minor global methylation pattern differences and a trend toward hypomethylation in ZIKV-positive cells, clear-cut evidence of global divergence was lacking. Nevertheless, each ZIKV-infected cell type harbored a substantial contingent of specific hypomethylated and a relatively smaller set of hypermethylated DMRs as determined by hierarchical clustering (Fig. 2E) (weighted *t* test, *q* value of <0.050, with an average methylation difference of >0.2). To focus on methylation changes that could influence gene expression, we next selected DMRs near transcription start sites (TSSs; 5,000 bp upstream to 500 bp downstream) for further analysis, which revealed a 30 to 40% excess of hypomethylated regions (Fig. 3A and B; Table S1). This observation resonates with the results obtained from our whole-brain organoids indicating that ZIKV alters DNA methylation at specific gene loci.

10.1128/mSystems.00219-17.8 TABLE S1  Differentially methylated regions near transcription start sites (5,000 bp upstream to 500 bp downstream) of organoid-derived astrocytes, neurons, and neural progenitor cells. ZIKV^+^ sorted cells from ZIKV-infected cerebral organoid cultures are compared to cells sorted from mock-infected cerebral organoid cultures. Download TABLE S1, XLSX file, 0.1 MB. Copyright © 2018 Janssens et al. 2018 Janssens et al. This content is distributed under the terms of the Creative Commons Attribution 4.0 International license.

Next, we estimated the impact of DNA methylation changes on gene transcription by comparing the transcriptome sequencing (RNA-seq) signature of ZIKV-infected neuronal progenitors (5) with our DMR list and found approximately 13 to 17% overlap. Independent quantitative reverse transcription-PCR (RT-PCR) then confirmed the expected transcriptional effects in 7 of 10 selected cases (Fig. S7).

Finally, we exposed pure human ESC-derived neural progenitors and hiPSC-derived astrocytes and neurons to ZIKV and mapped the methylation deviations (Fig. S6A and Table S2). Although the results clearly showed that ZIKV can alter the methylome, the impact of infection was attenuated compared to findings in organoid-derived cells (Fig. S6B to D). These results suggest that the response to the virus depends, in part, on the physiologic microenvironment of the cells. Thus, the impact of ZIKV on DNA methylation does not appear to be cell autonomous but rather might reflect a blending of the intrinsic cellular effects with secondary effects from heterogeneous cell-cell communication with neighboring cells. A subtle but possibly important contributing factor to the divergence seen between the two systems might also be an intrinsic variance in cell states as it arises as a consequence of the different differentiation environments. It can be anticipated that upon ZIKV-induced neuroinflammation, reactive astrocytes release cytokines and chemokines, and stressed or injured neurons will emit danger signals as part of an innate immune reaction (19).

10.1128/mSystems.00219-17.6 FIG S6  Identification of ZIKV-induced DNA methylation changes in pure human ESC-derived NPCs and iPSC-derived astrocytes and neurons; correlation of the differentially methylated genes with psychiatric disorders. (A) Scheme of sample generation used in RRBS analysis. (B) Global distribution of methylation levels in samples as indicated. (C) Principal-component analysis based on the mean methylation levels of 100-bp tiles. (D) Heat maps of differentially methylated 100-bp tiles of each cell type (*q* value of <0.05 and methylation difference of >0.2). (E) Dot plot of DisGeNET diseases showing correlation with differentially methylated gene loci (500 bp downstream to 5,000 bp upstream of transcriptional start site) (astro, astrocytes; neuro, neurons; npc, neural progenitor cells). (F) Distribution of genes associated with PsyGeNET disease categories according to cell type (UD, use disorders). Download FIG S6, DOCX file, 0.6 MB. Copyright © 2018 Janssens et al. 2018 Janssens et al. This content is distributed under the terms of the Creative Commons Attribution 4.0 International license.

10.1128/mSystems.00219-17.7 FIG S7  ZIKV-induced DNA methylation changes in cerebral organoid-derived neural progenitor cells are associated with transcriptional changes. (A) Venn diagram showing numbers of hypomethylated (hypo) and hypermethylated (hyper) gene loci in ZIKV (MR766)-infected organoid-derived neural progenitor cells and overlap with genes that are up- or downregulated as shown in RNA-seq analyses of ZIKV (MR766)-infected human iPSC-derived cortical neural progenitors (5). (B) Quantitative RT-PCR of selected genes in organoid-derived neural progenitors infected with ZIKV strains MR766 and PR as indicated. Shown are gene expression levels of genes that were identified in panel A as upregulated and hypomethylated (top) or downregulated and hypermethylated (bottom). Download FIG S7, DOCX file, 0.1 MB. Copyright © 2018 Janssens et al. 2018 Janssens et al. This content is distributed under the terms of the Creative Commons Attribution 4.0 International license.

10.1128/mSystems.00219-17.9 TABLE S2  Differentially methylated regions near transcription start sites (5,000 bp upstream to 500 bp downstream) of pure astrocytes, neurons, and neural progenitor cells. ZIKV^+^ cells sorted from ZIKV-infected pure cell cultures are compared to mock-infected cells. Download TABLE S2, XLSX file, 0.1 MB. Copyright © 2018 Janssens et al. 2018 Janssens et al. This content is distributed under the terms of the Creative Commons Attribution 4.0 International license.

### ZIKV-induced DMRs are linked to neurodevelopmental and psychiatric disorders.

To relate the DMRs caused by ZIKV infection to neurologic disease, we began by searching for a gene signature that might define microcephaly, the hallmark birth defect associated with the virus. This analysis identified 53 genes in the proximity of DMRs that were significantly related to a microcephalic phenotype (Fig. 3C) (*P* = 0.001 by Fisher’s exact test), attesting to the strength of our methylome analysis in the cerebral organoid model.

Apart from microcephaly, ZIKV has been linked to conditions such as cerebral palsy, intellectual disabilities, and epilepsy (20). Because prenatal virus-like immune activation can trigger stable DNA methylation changes in mouse brains that can underlie behavioral and cognitive deficits in offspring (8), we asked if our DMRs could be related to neurological diseases as well. Thus, we used the DisGeNET disease ontology algorithm (21) to examine all DMRs identified near transcription start sites (Fig. 3A). The top-ranked disease categories (Fig. 3D) included conditions such as mental retardation as well as intellectual and developmental disorders that showed strong correlations in at least two of the three cell types that we investigated. There were further associations of affected gene loci in astrocytes and neural progenitors with epilepsy and attention deficit hyperactivity disorder, respectively, consistent with published reports in which astrocytic and neuronal impairments have been associated with these conditions (22, 23). Our analysis also revealed correlations with craniofacial abnormalities and congenital facial anomalies, as well as FG syndrome and Aicardi syndrome, which are characterized by the partial or complete absence of the corpus callosum, a condition that has been described together with microcephaly in congenital ZIKV syndrome (24, 25). Similar, though less explicit, associations were apparent from our DisGeNET analysis of DMRs in pure cell populations (Fig. S6E).

**FIG 4:**
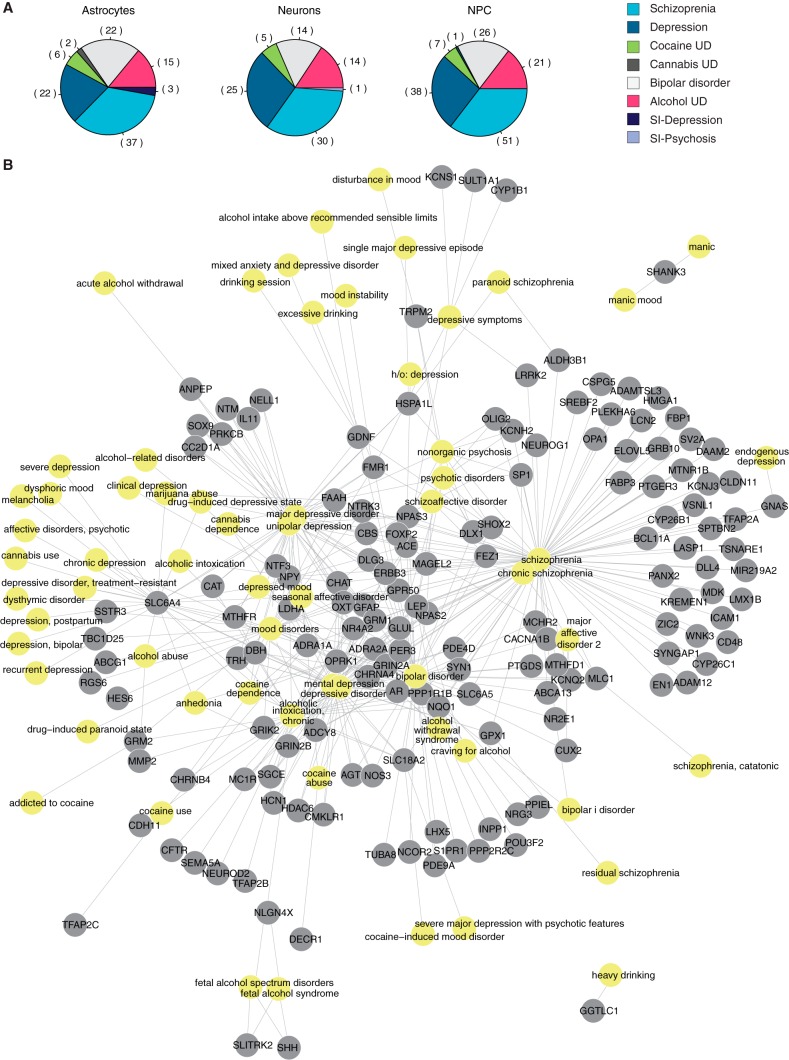
Correlation between neuropsychiatric disorders and genes affected by ZIKV-induced methylation changes. (A) Distribution of genes that associate with PsyGeNET disease categories according to cell type (SI, substance induced; UD, use disorders). (B) Analyses of gene networks and their association with psychiatric diseases. Included are all genes that were identified in at least one of the cerebral organoid-derived cell types. Yellow nodes represent diseases; gray nodes represent genes.

Especially provocative were the links between differentially methylated genes and neuropsychiatric disorders, in agreement with reports where other fetotrophic viruses were also thought to induce these diseases (26–28). To pursue this lead further, we reexamined our DMR data using PsyGeNET, a platform designed to reveal genetic links to eight classes of psychiatric disorders (29) (Fig. 4 and S6F). As many as 10% of the genes in the pool used to generate Fig. 3 were implicated in susceptibility to schizophrenia, bipolar illness, or other diseases covered by this platform (Fig. 4 and S6F). This finding indicates that microcephaly and other gross brain abnormalities may be only the tip of the iceberg in babies born after gestational ZIKV infection.

## DISCUSSION

A substantial proportion of the babies with ZIKV infection who were born with anatomic brain abnormalities can be expected to develop cognitive, behavioral, and mental health problems later in life and therefore are beginning to be closely monitored for these complications (30). Of growing concern are the infants who have been exposed to ZIKV but lack any clinical evidence of the infection at birth. Our findings predict that many of these individuals may have changes in their DNA methylome that could ultimately affect the expression of key genes involved in a spectrum of neuropsychiatric disorders. Our findings, together with recent clinical reports describing a causal relationship between ZIKV infection in adolescents and severe depression (31) and psychosis (32), support the hypothesis that ZIKV might directly trigger neuropsychiatric and cognitive disease. Hence, while more is being learned about the consequences of severe symptomatic ZIKV infection, our results reinforce the concern that asymptomatic prenatal ZIKV infections could have long-term effects on neurodevelopment, justifying further rigorous study to identify infected individuals and mechanisms of disease induction.

We show in this report that ZIKV alters DNA methylation in developing brain cells. Although the virus dampens DNA methyltransferase activity (5, 33), how it globally alters specific DMRs remains unexplained and should shift our focus toward the direct action of ZIKV proteins on chromatin. The observation that methylation changes are more profound in a heterocellular organoid-derived environment than in pure neuronal cells suggests that not only the virus itself but also the entire cellular and immunoreactive milieu contributes to the effects on DNA methylation. It remains open, however, whether the methylation changes come first or whether the viral infection dysregulates epigenetic regulatory genes prior to any epigenetic shift.

Although the Zika virus can directly eliminate developing brain cells (3, 5), the altered DNA methylation pattern in surviving cells has the potential to drive a range of neurological symptoms in babies born to infected mothers. It is interesting that 94% of the Zika virus-related cases of microcephaly identified in a recent Brazilian outbreak occurred in the most economically depressed region of northern Brazil (1); hence, an inadequate diet might render the fetus more vulnerable to methylation changes. If so, nutritional supplementation could afford effective prophylaxis, a prediction requiring validation in future *in vitro* and animal studies. Furthermore, different human genetic ancestries shape the methylome in unique ways in response to environmental factors such as tobacco smoke (34), and it may not come as a surprise if ethnic groups show *sui generis* epigenetic patterns that translate into variable susceptibilities to ZIKV infection. In that context, it appears noteworthy that the H9 embryonic stem cells that we used to make brain organoids possess Caucasian ethnicity (35). Thus, it will be interesting to see if infection of organoids derived from cells of Brazilian genetic background might show even more severe methylation and gene expression changes related to microcephaly or other neurological diseases and if nutritional supplements can interfere with such changes.

Despite the fact that ZIKV first affected the African continent (African ZIKV lineage) before its jump to Micronesia and widespread transmission in the Americas (Asian ZIKV lineage), the issue of congenital infection and causality with microcephaly was discovered only during the outbreak in Brazil. Many factors specific to the Brazilian population have been proposed to explain this phenomenon, including socioeconomics, variability in host genetics, virus strain-specific modifications, and cross-reaction of antibodies to dengue virus with ZIKV that would result in intensified ZIKV-related symptoms such as microcephaly by facilitating transplacental spread of the virus (36–38). However, a World Health Organization ZIKV situation report, published in September 2016, described a number of microcephaly cases in Guinea-Bissau, West Africa, that might have been caused by *in utero* exposure to an African Zika virus lineage strain (39). Many similarities, but also some experimental differences, have been reported for the African and Asian lineage ZIKV strains (40, 41). In the work described here, we performed experiments with the African lineage MR766 ZIKV strain and included additional infection with the Asian lineage PRVABC59 strain in some of the experiments. Although, upon infection with the two strains, similar conclusions could be made in our experiments, we cannot rule out the possibility that some of the observations are specific to the African lineage MR766 ZIKV strain, and so it will be intriguing to learn if different ZIKV strains could influence the epigenetic impact of ZIKV infection. Until further studies are performed, our results are most consistent with the hypothesis that prenatal ZIKV infection could have extensive, long-term postinfectious consequences for aberrant neurodevelopment.

## MATERIALS AND METHODS

### Human cerebral organoid, neural stem cell, cortical neuron, and astrocyte cultures.

Human H9 embryonic stem (WA09) cells were obtained from WiCell and maintained with standard protocols in mTESR1 (Stem Cell Technologies). Cerebral organoids were generated and cultured in a bioreactor according to a published protocol (42), except that Aggrewell 800 plates (Stem Cell Technologies) were used for embryonic body formation. Prior to infection, cerebral organoids were dissociated with Accutase (Innovative Cell Technologies) at 37°C for 10 min and grown for 7 days on growth factor-reduced Matrigel-coated culture dishes (Corning) in cerebral organoid differentiation medium. Cultures were fed every 3 to 4 days by replacing half of the medium.

Gibco H9 (WA09)-derived neural stem cells were cultured according to the manufacturer’s recommendations. Briefly, cells were cultured as a monolayer on poly-l-ornithine (Sigma)- and laminin (Invitrogen)-coated culture dishes in StemPro NSC serum-free complete medium (cells and culture reagents were obtained from Thermo Fisher). Culture medium was changed every 2 days, and cells were passaged at 90% confluence with TrypLE (for a maximum of 3 passages).

iCell hiPSC-derived human cerebral cortical neurons and hiPSC-derived human astrocytes were acquired from Cellular Dynamics International (CDI) and handled according to the manufacturer’s recommendations. Cerebral cortical neurons were seeded at a density of 1.25 × 10^5^ cells/cm^2^ on poly-l-ornithine- and laminin-coated culture dishes in complete maintenance medium (CDI); complete medium changes were made after 24 h *in vitro*, and half of the medium was changed every 3 to 4 days. Astrocytes were seeded at a density of 5.5 × 10^4^ cells/cm^2^ on growth factor-reduced Matrigel-coated culture dishes (Corning) in astrocyte medium: Dulbecco’s modified Eagle’s medium (DMEM), high glucose, GlutaMAX, and pyruvate (Life Technologies) with 1× N-2 supplement (Life Technologies) and 10% fetal bovine serum (FBS; HyClone). Cells were given complete medium changes after 24 h *in vitro* and subsequently fed every 2 to 3 days by replacing half of the medium. The cells were passaged once with TrypLE, when they reached 90% confluence.

### Immunofluorescence staining.

Dissociated organoid cultures were grown on growth factor-reduced Matrigel (Corning)-coated chambered coverslips and fixed with 4% paraformaldehyde (PFA) in phosphate-buffered saline (PBS) for 10 min at room temperature. Whole cerebral organoids were fixed in 4% PFA and processed for cryosectioning (42). For immunofluorescence staining, sections or cells were immunoblocked and permeabilized in 0.25% Triton X-100 and 4% goat serum in PBS followed by overnight incubation with primary antibodies in 0.1% Triton X-100 and 4% goat serum in the following dilutions: TUJ1 (mouse; BioLegend catalog no. 801201), 1:500; PAX6 (rabbit; BioLegend catalog no. 901301), 1:100; glial fibrillary acidic protein (GFAP) (rabbit; Dako catalog no. Z0334), 1:500; doublecortin (DCX) (guinea pig; Invitrogen catalog no. A-11075), 1:500; anti-flavivirus group E antigen 4G2 (mouse; Millipore; catalog no. MAB10216), 1:200; anti-flavivirus NS5 (chicken, made in-house [14]), 1:500. Secondary antibodies consisted of Alexa Fluor 488, rabbit Alexa Fluor 594, chicken Alexa Fluor 555, and guinea pig Alexa Fluor 568 conjugates (Invitrogen) and were used in dilutions of 1:200. Images were taken with a Leica SP5 DMI inverted confocal microscope.

### Virus production.

Zika virus strains Uganda 1947 (MR766) and Puerto Rico 2015 (PRVABC59) were grown on monolayers of Vero cells in Dulbecco’s modified Eagle’s medium supplemented with 2% fetal calf serum and penicillin-streptomycin (Sigma) in T175 culture flasks. After 72 h, the culture supernatant was collected and centrifuged (400 × *g*, 10 min) to remove cellular debris. The resultant virus suspension was aliquoted and stored at −80°C until further use. Previous work (40) confirmed that the MR766 strain used does not contain the reported mutations, including 4 to 6 codon deletions within the E protein, acquired by serial passaging in mouse brains (43).

### Manual antibody conjugation.

One hundred micrograms of purified 4G2 antibody (Millipore; catalog no. MAB10216) or in-house-produced and purified 4G2 antibody (36) was conjugated to Alexa Fluor 488 with the Invitrogen antibody labeling kit (catalog no. A20181) according to the protocol provided by the manufacturer.

### Fluorescence-activated cell sorting strategy.

Organoid-derived cell mixtures were stained for the extracellular markers CD184, CD44, CD24, CD271, and CD15 to separate neural progenitors, astrocytes, and neurons as described by Yuan et al. (17). Cell monolayers were dissociated with Accutase (Innovative Cell Technologies), washed in PBS with 1% bovine serum albumin (BSA) and 2 mM EDTA, and stained with the directly conjugated surface marker antibodies for 45 min at room temperature in the dark (V450-conjugated anti-human CD15 [BD Biosciences; catalog no. 561584], BUV395-conjugated anti-human CD24 [BD Biosciences; catalog no. 563818], peridinin chlorophyll protein [PerCP]-Cy5.5-conjugated anti-human CD44 [BD Biosciences; catalog no. 560531], allophycocyanin [APC]-conjugated anti-human CD184 [BD Biosciences; catalog no. 555976], and phycoerythrin [PE]-conjugated anti-human CD271 [BD Biosciences; catalog no. 557196]). To distinguish ZIKV-infected from uninfected cells, cells were washed, fixed with 2% paraformaldehyde for 5 min, permeabilized in PermWash buffer (BD Biosciences), washed, and stained for 1 h with the in-house-conjugated Alexa Fluor 488-conjugated monoclonal anti-flavivirus group antigen antibody (clone 4G2). Cells were washed once in PermWash buffer and resuspended in PBS with 1% BSA for subsequent FACS or flow cytometry. Gates were first applied to isolate single cells, after which they were sorted for CD271 and CD44. CD271^−^ CD44^+^ cells were then further gated for CD184^+^ to obtain cells with the astrocyte marker signature. CD271^−^ CD44^−^ cells were further gated for CD184, CD24, and CD15 to separate neural progenitor cells (CD271^−^ CD44^−^ CD184^+^ CD24^+^) and neurons (CD271^−^ CD44^−^ CD184^−^ CD24^+^ CD15^low^).

### RNA extraction and quantitative RT-PCR.

RNA was extracted from thawed cell pellets with the Direct-zol RNA miniprep kit (Zymogen) including DNase treatment according to the manufacturer’s instructions. The WTA2 kit (Sigma) was used to generate a cDNA library according to the protocol provided by the manufacturer (25 cycles), and PCR was performed with the PerfeCTa SYBR green FastMix (Quanta Biosciences) using a Roche LightCycler 480.

### DNA extraction.

Cell pellets were thawed and resuspended in 300 µl lysis buffer (10 mM Tris, pH 8.0, 10 mM EDTA, 10 mM NaCl, 0.5% SDS, 1 mg/ml proteinase K, 50 µg/ml DNase-free RNase A) at 55°C overnight. DNA was extracted with phenol-chloroform followed by NaCl-ethanol precipitation. Genomic DNA was washed with 70% ethanol, air dried, and resuspended in 20 µl EB buffer (10 mM Tris-HCl, pH 8.5, 0.1 mM EDTA).

### WGBS library construction.

Genomic DNA (200 ng) was fragmented with a Covaris S2 sonicator for 6 min with the 5% duty cycle, an intensity of 5, and 200 cycles per burst. The sheared DNA was purified with the DNA Clean and Concentrator kit from Zymo Research according to the manufacturer’s recommendations. Bisulfite conversion of DNA was then conducted with the EZ DNA Methylation-Gold kit (Zymo Research) according to the manufacturer’s protocol and eluted in 15 µl low-Tris-EDTA (TE) buffer. The converted DNA was immediately processed, and WGBS libraries were generated using the Accel-NGS Methyl-Seq DNA library kit (Swift Biosciences) according to the manufacturer’s protocol. The libraries were sequenced for 100-bp paired-end reads on an Illumina HiSeq 2500 sequencer.

### RRBS and WGBS data processing and analysis.

Raw sequencing reads were trimmed by 10 bp at each end and aligned to the human genome build hg19/GRCh37 using BSMAP (44). Methylation levels at CpGs were determined with the mCall package from MOABS (45). Only CpGs covered by 5 or more reads were used for analysis. DMRs were identified by a two-sample weighted *t* test, and multiple-testing correction was performed with the R *q* value package. For the 1-kb tiles, at least 5 CpGs were required to be covered at 5×. For the 100-bp tiles, at least 2 CpGs were required to be covered at 5×. The R MethyAnalysis with the TxDb.Hsapiens.UCSC.hg19.knownGene package was used to associate genes with DMRs. The R DOSE (46) package was used for disease ontology analyses. A background gene list for enrichment analyses was generated by subjecting all defined tiles of the genome to the same annotation criteria as the DMRs. To determine if ZIKV-induced methylation changes target distal enhancer elements, we aligned the 818 identified DMRs with 1,837 curated human enhancers available through the VISTA enhancer browser (https://enhancer.lbl.gov/). For psychiatric disease association analyses, the R psygenet2r package was used. The human phenotype ontology gene set for microcephaly was retrieved from the HPO browser (http://compbio.charite.de/hpoweb/showterm?id=HP:0000252). RNA-seq data for ZIKV-infected hiPSC-derived cortical neural progenitors were retrieved from the work of Tang et al. (5); epigenetic data for fetal brain were obtained from the NIH Epigenomics Roadmap Consortium (47).

### Accession number(s).

All data have been deposited in the Gene Expression Omnibus (GEO) under accession number GSE109104.
