# Supplementary material for: Zika Virus Alters DNA Methylation of Neural Genes in an Organoid Model of the Developing Human Brain
Source: mSystems. 2018 Feb 6;3(1):e00219-17. doi: 10.1128/mSystems.00219-17 (PMC5801341; doi:10.1128/mSystems.00219-17)
Supplement: FIG S1 [file sys001182169sf1.docx]

**Figure S1. Human ESC-derived cerebral organoid culture and detection of astrocytes, neurons and neural progenitor cells.** (**A**) Phase contrast or bright-field (day 96) images of developing cerebral organoids. Different organoids are shown at different time points. Scale bar = 1000 µm. (**B**) Phase contrast microscopy of a 2D culture of 112 day-old organoids 5 days after dissociation shows different types of neuronal cells. Scale bar = 50 µm. (**C**) Immunofluorescence staining to detect astrocyte (GFAP, red), neuron (TUJ1, green) and neural progenitor (PAX6, red) markers in 16 week-old organoids. Scale bar = 100 µm. (**D**) Immunofluorescence staining to detect astrocyte (GFAP, red), neuron (DCX, red) and neural progenitor (PAX6, red) markers in organoid-derived dissociated multicellular cultures. Scale bar = 50 µm.
