# Supplementary material for: Zika Virus Alters DNA Methylation of Neural Genes in an Organoid Model of the Developing Human Brain
Source: mSystems. 2018 Feb 6;3(1):e00219-17. doi: 10.1128/mSystems.00219-17 (PMC5801341; doi:10.1128/mSystems.00219-17)
Supplement: FIG S2 [file sys001182169sf2.docx]

**Figure S2. The 1947 Uganda and 2015 Puerto Rico ZIKV strains show comparable infection rates in human ESC-derived cerebral organoid cultures.** (**A**) Immunofluorescence staining to detect the flaviviral E- and NS5-protein in multicellular 2D cultures derived from 84d-old organoids infected with the Uganda (MR766) or Puerto Rico (PR) ZIKV strains. Scale bar = 10 µm. (**B**) Infection rates of 16‑week-old cerebral organoid derived astrocytes (astro), neural progenitor cells (NPC) and neurons (neuro) upon infection with the MR766 or PR ZIKV strains as determined by flow cytometry in two independent experiments.
