# Supplementary material for: Zika Virus Alters DNA Methylation of Neural Genes in an Organoid Model of the Developing Human Brain
Source: mSystems. 2018 Feb 6;3(1):e00219-17. doi: 10.1128/mSystems.00219-17 (PMC5801341; doi:10.1128/mSystems.00219-17)
Supplement: FIG S3 [file sys001182169sf3.docx]

**Figure S3. ZIKA sensitive DNA regions align well with regions of the fetal brain that are methylated.** Visualization of methylation sensitive restriction enzyme sequencing (MRE-seq) data obtained from fetal brain tissue at 17 weeks of gestation (NIH Epigenomics Roadmap Consortium, samples HuFNSC01, HuFNSC02) aligned with ZIKV induced differentially methylated regions (DMRs) identified in cerebral organoids by WGBS (1kb tiles) in our study. Shown is the alignment of all human autosomes and the X chromosome (all analyzed samples are female).
