# Supplementary material for: Zika Virus Alters DNA Methylation of Neural Genes in an Organoid Model of the Developing Human Brain
Source: mSystems. 2018 Feb 6;3(1):e00219-17. doi: 10.1128/mSystems.00219-17 (PMC5801341; doi:10.1128/mSystems.00219-17)
Supplement: FIG S4 [file sys001182169sf4.docx]

**Figure S4. Illustration of epigenetic marks in fetal brain gene regions that are ZIKV sensitive.** Fetal brain epigenetic marks (at 17 weeks of gestation, NIH Epigenomics Roadmap Consortium) are shown for 200-250kb region around the (**A**) hypomethylated DDX3X locus, and the (**B**) hypermethylated IRX3 locus in ZIKV infected cerebral organoids (see also Figure 1D). DMRs, differentially methylated regions; Gray, areas with non-significant sequencing signal; red marks, differentially methylated regions.
