# Supplementary material for: Zika Virus Alters DNA Methylation of Neural Genes in an Organoid Model of the Developing Human Brain
Source: mSystems. 2018 Feb 6;3(1):e00219-17. doi: 10.1128/mSystems.00219-17 (PMC5801341; doi:10.1128/mSystems.00219-17)
Supplement: FIG S5 [file sys001182169sf5.docx]

**Figure S5. FACS strategy to segregate human ESC-derived cerebral organoids into astrocytes, neurons and neural progenitor cells.** (**A**) Examples of cell sorting plots and marker signatures (table) used to isolate astrocytes, neurons and neural progenitor cells (NPC) from human ESC derived cerebral organoid cultures. (**B**) Examples of FACS gating and percentages of ZIKV-infected cells among isolated astrocytes, neurons and, NPCs from infected (MR766) multicellular cerebral organoid cultures. (**C**) Scatterplot showing the distribution of astrocytes, neurons, and NPCs in ZIKV-infected (MR766) cerebral organoids in three independent experiments. The overall trend favors the presence of astrocytes (astro) and neural progenitors (NPC) over mature neurons (neuro) in the ZIKV+ cell fraction.
