# Supplementary material for: Zika Virus Alters DNA Methylation of Neural Genes in an Organoid Model of the Developing Human Brain
Source: mSystems. 2018 Feb 6;3(1):e00219-17. doi: 10.1128/mSystems.00219-17 (PMC5801341; doi:10.1128/mSystems.00219-17)
Supplement: FIG S6 [file sys001182169sf6.docx]

**Figure S6. Identification of ZIKV induced DNA methylation changes in pure human ESC-derived NPCs and iPSC-derived astrocytes and neurons; correlation of the differentially methylated genes with psychiatric disorders.** (**A**) Scheme of sample generation used in RRBS analysis. (**B**) Global distribution of methylation levels in samples as indicated. (**C**) Principal component analysis based on the mean methylation levels of 100‑bp tiles. (**D**) Heatmaps of differentially methylated 100‑bp tiles of each cell type (q‑value < 0.05 and methylation difference > 0.2). (**E**) Dot plot of DisGeNET diseases showing correlation with differentially methylated gene loci (500 bp downstream to 5000 bp upstream of TSS) (astro, astrocytes; neuro, neurons; NPC, neural progenitor cells; TSS, transcriptional start site). (**F**) Distribution of genes associated with PsyGeNET disease categories according to cell type (UD, use disorders).­­
