# Supplementary material for: Zika Virus Alters DNA Methylation of Neural Genes in an Organoid Model of the Developing Human Brain
Source: mSystems. 2018 Feb 6;3(1):e00219-17. doi: 10.1128/mSystems.00219-17 (PMC5801341; doi:10.1128/mSystems.00219-17)
Supplement: FIG S7 [file sys001182169sf7.docx]

**Figure S7. ZIKV induced DNA methylation changes in cerebral organoid derived neural progenitor cells are associated with transcriptional changes.** (**A**) Venn diagram showing numbers of hypomethylated (hypo) and hypermethylated (hyper) gene loci in ZIKV (MR766) infected organoid derived neural progenitor cells and overlap with genes that are up- or downregulated as shown in RNA-seq analyses of ZIKV (MR766) infected human iPSC derived cortical neural progenitors (Tang et al., 2016). (**B**) qRT-PCR of selected genes in organoid-derived neural progenitors infected with ZIKV strains MR766 and PR as indicated. Shown are gene expression levels of genes that were identified in (A) to be upregulated and hypomethylated (top) or downregulated and hypermethy­­­lated (bottom). ­­
